# Histological concordance of medial arterial calcification between tibial and peroneal arteries in advanced peripheral arterial disease

**DOI:** 10.1016/j.jvssci.2026.100426

**Published:** 2026-05-15

**Authors:** Snow Adler, Jorge RuizdelRio, Brian Eliceiri, Ann C. Gaffey

**Affiliations:** a Department of Surgery, Naval Medical Center San Diego, San Diego, CA; b Department of Surgery, University of California San Diego, La Jolla, CA; c Department of Vascular and Endovascular Surgery, University of California San Diego, La Jolla, CA

**Keywords:** Medial arterial calcification, Critical limb-threatening ischemia, Peripheral artery disease, Alizarin Red, Tibial artery, Peroneal artery

## Abstract

**Background:**

Medial arterial calcification (MAC) is prevalent in peripheral artery disease (PAD), yet its distribution and clinical significance in infrapopliteal arteries remain poorly defined. Prior histopathological studies of distal tibial segments have not evaluated MAC as a vessel-level lesion. Peroneal arteries (PAs) are frequently regarded as relatively spared revascularization targets based on angiographic assessment alone, despite limited histological validation and emerging evidence that arterial medial remodeling contributes independently to limb ischemia. Accordingly, it remains unclear whether MAC in PAD reflects a diffuse distal arterial phenotype shared across tibial and peroneal segments or a segment-specific process.

**Methods:**

This single-center prospective pilot study included 14 patients and 14 limbs (13 patients with PAD and 1 control without PAD) undergoing below-knee amputation. Paired anterior tibial arteries (ATAs) and PAs were harvested, formalin fixed, and paraffin embedded. Hematoxylin and eosin staining was used to assess arterial architecture, intimal plaque, and medial remodeling. MAC was graded 0 to 4 using Alizarin Red staining according to circumferential extent and thickness. Clinical variables included age, sex, ethnicity, diabetes mellitus (DM), hemoglobin A1c, end-stage renal disease stage, smoking status, glucagon-like peptide-1 receptor agonist use, Rutherford category, and limb outcomes.

**Results:**

The cohort included 92.3% males with an average age of 69.0 ± 10 years and a high prevalence of DM, consistent with larger PAD series. Moderate to severe (grades 3-4) MAC was common in limbs with PAD. Within-patient concordance of MAC grades between paired ATA and PA was high, with most pairs differing by ≤1 grade, supporting a diffuse rather than a segment-specific distal arterial phenotype. A greater MAC burden was observed in patients with DM, advanced end-stage renal disease, and higher Rutherford categories, but these exploratory associations were not statistically significant. Directional trends were observed between histological MAC burden and adverse limb outcomes, consistent with prior radiographic studies.

**Conclusions:**

In this pilot human tissue study, MAC appears to represent a shared distal arterial phenotype across ATAs and PAs in PAD rather than a segment-specific lesion. Histology-confirmed MAC burden provides biologic validation of distal calcification patterning and may help to refine our understanding of infrapopliteal disease in advanced PAD. Larger, multicenter studies are warranted to define the prognostic utility of MAC-based indices and to determine how tissue-level calcification patterns relate to clinical imaging and revascularization strategies.

**Clinical relevance:**

In advanced peripheral artery disease, medial arterial calcification (MAC) appears to be a shared distal arterial phenotype rather than a lesion confined to a single infrapopliteal vessel. The high concordance between paired anterior tibial artery and peroneal artery specimens suggests that the histological assessment of one distal artery may reflect limb-wide MAC burden, which could help to refine the risk stratification and interpretation of infrapopliteal disease. These findings also support the concept that the peroneal artery is not histologically spared in severe peripheral artery disease, despite its frequent consideration as a revascularization target. Larger studies integrating tissue histology, imaging, and clinical outcomes are needed to determine whether MAC burden can improve prediction of limb loss and guide procedural planning.

**Article Highlights**

• **Type of Research:** Human study
• **Key Findings:** Paired anterior tibial and peroneal arteries in 13 patients with peripheral artery disease (PAD) and 1 patient without PAD showed high histological concordance in medial arterial calcification in advanced PAD. Medial arterial calcification appeared to reflect a shared distal arterial phenotype rather than a vessel-specific lesion, with many limbs showing moderate to severe calcification and exploratory links to diabetes, end-stage renal disease, and worse limb severity.
• **Take Home Message:** Medial arterial calcification in advanced PAD is a shared distal phenotype across paired anterior tibial and peroneal arteries, so the calcification burden is broadly concordant within the limb rather than confined to one vessel.

Peripheral artery disease (PAD) affects an estimated 230 million adults worldwide and represents a leading cause of lower extremity amputation and cardiovascular morbidity.^1^ Chronic limb-threatening ischemia, the most severe manifestation of PAD, is characterized by rest pain, tissue loss, or gangrene, and is associated with major amputation rates of >25% at 1 year and mortality rates comparable with many solid organ malignancies.^2^ Prevention of major amputation requires successful revascularization. Yet, outcomes of endovascular therapy in infrapopliteal arteries remain suboptimal, in part because the heterogeneity of below-knee arterial tibial disease is incompletely characterized.

Medial arterial calcification (MAC) is increasingly recognized as an active, regulated process that contributes to arterial stiffness, impaired distal perfusion, and adverse cardiovascular outcomes in high-risk populations, particularly those with diabetes mellitus (DM) and chronic kidney disease.^3^, ^4^, ^5^, ^6^ Mechanistically, MAC shares fundamental features with osteogenesis, including osteogenic reprogramming of vascular smooth muscle cells, loss of calcification inhibitors such as matrix Gla protein, and deposition of hydroxyapatite mineral within the medial layer of the arterial wall.^3^, ^4^, ^5^, ^6^, ^7^ Unlike intimal calcification, which is closely associated with atherosclerotic plaque and luminal stenosis, MAC occurs preferentially within the tunica media and is not associated with lipid accumulation or inflammatory infiltrate, resulting in a distinct pathophysiological and clinical phenotype.^8^

Despite this mechanistic understanding, the distal limb vasculature in PAD has been relatively understudied at the histological level. Most clinical investigations rely on radiographic surrogates of MAC, mainly plain radiographic calcification scores.^2^^,^^9^, ^10^, ^11^ Radiographic studies demonstrate that calcification burden across vascular beds, including the infrapopliteal and pedal arteries, predicts cardiovascular mortality and major amputation independent of traditional risk factors. However, despite the prognostic value of tibial and pedal calcification scores, histological validation of MAC in human tissue remains lacking.^12^

A critical unresolved question is whether MAC in tibial arteries (TAs) vs peroneal arteries (PAs) represents segment-specific pathology, potentially driven by local hemodynamic factors, or a diffuse distal arterial phenotype reflecting systemic drivers such as DM and end-stage renal disease (ESRD).^1^ Prior histopathological comparisons of anterior TA (ATA) and posterior TA plaque characteristics identified differences in eccentric plaque morphology, macrophage infiltration, and thromboembolic burden, but did not specifically evaluate MAC as a vessel-level end point.^13^ Furthermore, the PA is frequently used as a target vessel for distal bypass or endovascular therapy given limited distal targets; however, the PA has not been characterized histologically with respect to MAC burden.^14^

In this pilot study, paired ATA and PA specimens from patients with and without advanced PAD were used to test the hypothesis that MAC is a diffuse distal arterial phenotype rather than a vessel-specific lesion. This study combined conventional histology and semiquantitative Alizarin Red grading to quantify MAC burden at the vessel level and to compare MAC distribution between paired TAs and PAs. By integrating these multimodal tissue measurements with detailed clinical phenotyping, this project aimed to establish a histology-based framework that links distal arterial calcification patterns to PAD severity. We hypothesized that MAC burden would be concordant between paired ATA and PA segments, supporting a shared distal arterial phenotype in advanced PAD.

## Methods

### Study population

This is a single-center prospective pilot study of adults undergoing below-knee amputation at our institution. Eligible patients included those with PAD undergoing amputation for chronic limb-threatening ischemia (Rutherford categories 4-6) and controls without PAD undergoing amputation for an oncologic or traumatic indication. Inclusion criteria required the availability of paired distal arterial tissues (ATA and PA) and the provision of written informed consent. Exclusion criteria included patients with active infection requiring staged amputations. This pilot study included 14 patients and 14 limbs (13 patients with PAD, 1 control without PAD); paired ATA and PA specimens were obtained from each limb for histological comparison. This study protocol was approved by the institutional review board, and all patients consented to deidentified examination of their arteries. Summarized in the Table are patient demographics and clinical characteristics, stratified by PAD vs non-PAD status.

**Table:** Patient demographics and clinical characteristics

Variables	Non-PAD (n = 1)	PAD (n = 13)
Age, years		
Mean ± SD	63.0 ± NA	69.0 ± 10.0
Range	63-63	52-87
Median	69-3.0	69.0
Sex		
Male	1 (100.0)	12 (92.3)
Female	0 (0.0)	1 (7.7)
Ethnicity		
Hispanic	0 (0.0)	10 (76.9)
White	1 (100.0)	3 (23.1)
Smoking status		
Current	1 (100.0)	0 (0.0)
Former	0 (0.0)	6 (46.1)
Never	0 (0.0)	7 (53.8)
DM		
Yes	0 (0.0)	12 (92.3)
No	1 (100.0)	1 (7.7)
HbA1c <5.7% (normal)	1 (100.0)	2 (15.4)
HbA1c 5.7%-6.4% (prediabetes)	0 (0.0)	3 (23.1)
HbA1c 6.5%-8.0% (controlled DM)	0 (0.0)	1 (38.5)
HbA1c >8.0% (poorly controlled DM)	0 (0.0)	1 (23.1)
GLP-1 RA Yes	0 (0.0)	4 (30.8)
GLP-1 RA No	1 (100.0)	9 (69.2)
Hypertension		
Yes	0 (0.0)	13 (100.0)
No	1 (100.0)	0 (0.0)
ESRD		
Yes	0 (0.0)	3 (23.1)
No	1 (100.0)	10 (76.9)
eGFR <60	0 (0.0)	3 (23.1)
eGFR ≥60	1 (100.0)	10 (76.9)
HD	0 (0.0)	3 (100.0)
PD	0 (0.0)	0 (0.0)
Rutherford class		
0	1 (100.0)	0 (0.0)
5	0 (0.0)	8 (61.5)
6	0 (0.0)	5 (38.5)
Time to amputation, days	27	112 ± 115

*DM*, Diabetes mellitus; *eGFR*, estimated glomerular filtration rate; *ESRD*, end-stage renal disease; *GLP-1 RA*, glucagon-like pepetide-1 receptor agonist; *HbA1c*, hemoglobin A1c; *HD*, hemodialysis; *NA*, not available; *PAD*, peripheral artery disease; *PD*, peritoneal dialysis; *SD*, standard deviation.

Continuous data are presented as mean ± SD or median (interquartile range), and categorical data as number (%).

Variables include age, sex, ethnicity, smoking status, and history of DM, hypertension, ESRD, and prior lower extremity intervention. Cohort of patients with DM was further categorized by HbA1c level and GLP-1 RA use. Patients with PAD was further categorized by Rutherford category.

Non-PAD group includes a single patient who is not diabetic and does not have ESRD. Due to the very small non-PAD sample (n = 1), no statistical tests or *P* values were calculated; data are descriptive.

### Specimen acquisition and processing

**Fig 1:**
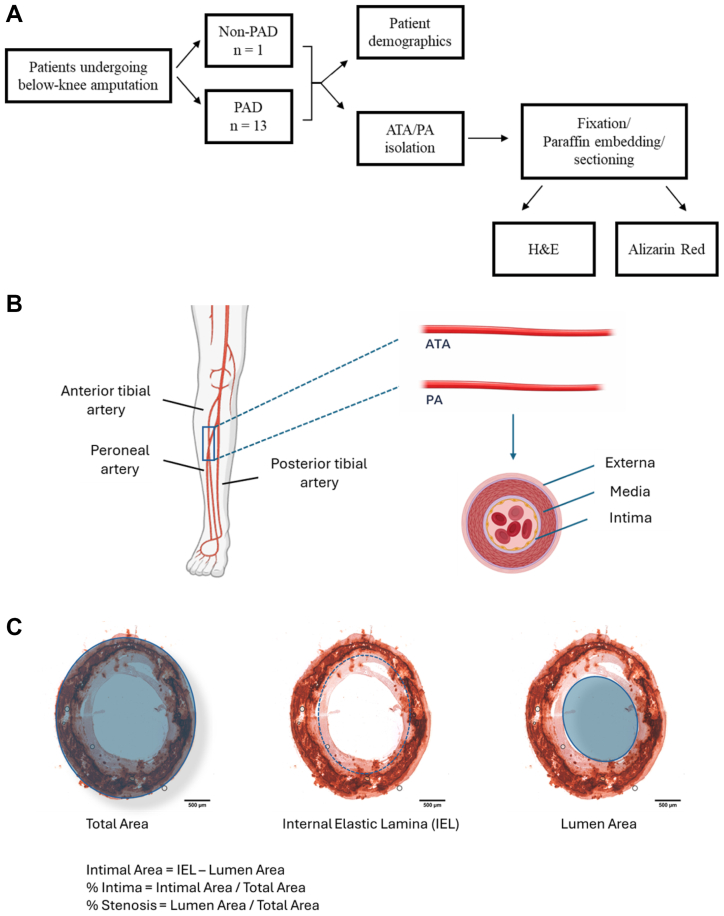
Patient and arterial specimen workflow. **(A)** Schematic illustrating patient enrollment, tissue harvesting, and multimodal histological processing for paired anterior tibial artery (*ATA*) and peroneal artery (*PA*) specimens. Distal artery tissue was obtained from patients undergoing below-knee amputation. peroneal artery. **(B)** For each patient, ATA and PA segments were harvested and sections from each vessel were processed and analyzed based on representative histological cross-sections. **(C)** Morphometric definitions for quantitative arterial histology. Schematic diagrams illustrate manual tracings used to define total vessel area, lumen area, and the internal elastic lamina (*IEL*). Intimal area was calculated as the IEL area minus the lumen area, percent intima as intimal area divided by total vessel area, and percent stenosis as lumen area divided by total vessel area. *H&E*, Hematoxylin and eosin; *PAD*, peripheral artery disease.

At the time of amputation surgery, paired ATAs and PAs were harvested from the amputated limb by the operating surgeon. Vessels were excised from approximately 5 cm distal to the amputation margin to approximately 5 cm proximal to the ankle; carefully dissected free of surrounding vein, nerve, and soft tissue; and flushed with 10 mL of heparinized saline within 20 minutes of amputation. The average harvested vessel length was 12 cm for the ATA and 6 cm for the PA. Specimens were immediately rinsed with phosphate-buffered saline, fixed in 10% neutral buffered formalin for 24 to 48 hours, and processed into formalin-fixed paraffin-embedded blocks using standard histological protocols. Each vessel was sectioned transversely at approximately 5-mm intervals to generate representative cross-sectional tissue sections. The overall patient and arterial specimen workflow is illustrated in Fig 1.

### Histology and staining

**Fig 2:**
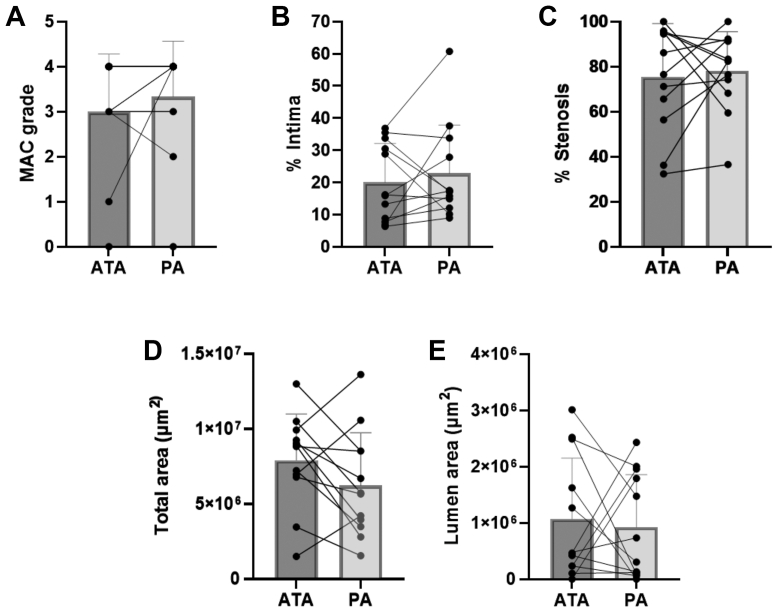
Paired anterior tibial artery (*ATA*) and peroneal artery (*PA*) histological and morphometric comparisons within individual patients. **(A)** Semiquantitative medial arterial calcification (*MAC*) grade, **(B)** percent intima, **(C)** percent stenosis, **(D)** total vessel area, and **(E)** lumen area are shown for paired ATA and PA specimens from each patient. Each line connects matched ATA and PA values from the same limb, highlighting within-patient concordance and vessel-to-vessel variation.

Hematoxylin and eosin staining was performed on all sections to assess the overall arterial architecture, intima-media delineation, and smooth muscle cell morphology. Comparative vessel-specific histological parameters are summarized in Fig 2.

**Fig 3:**
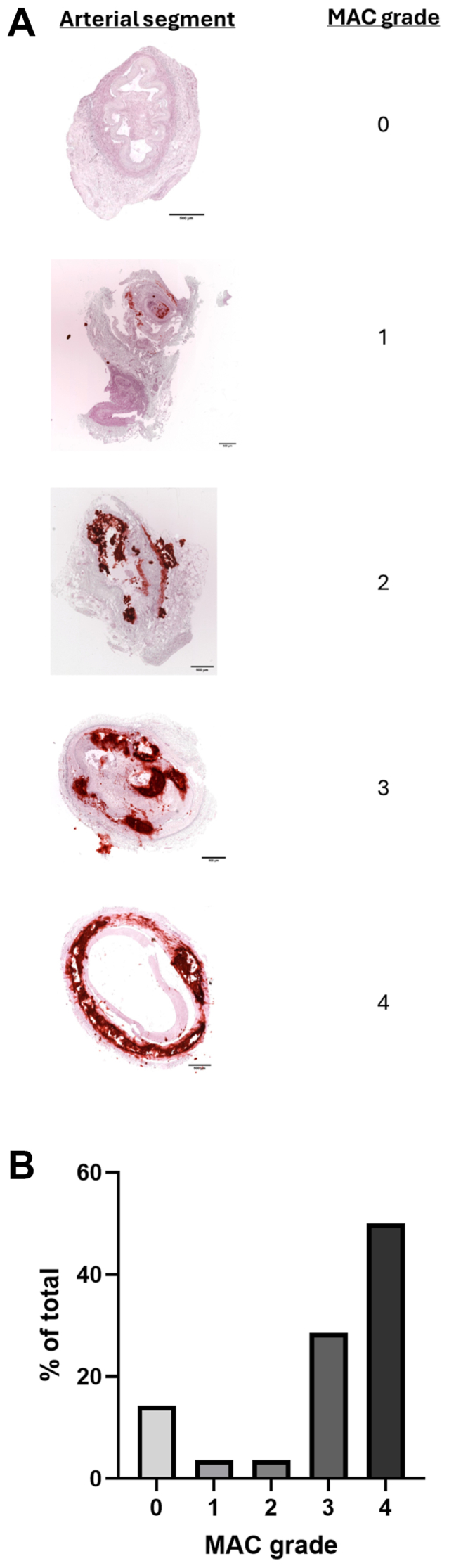
Alizarin Red semiquantitative grading scale for medial arterial calcification (*MAC*) and representation of patient samples. **(A)** Representative photomicrographs of anterior tibial artery (ATA) and peroneal artery (PA) cross-sections demonstrating MAC grade 0 (no calcification) through grade 4 (near-circumferential dense medial calcification). Alizarin Red staining; scale bar, 500 μm. **(B)** Distribution of MAC grades in paired anterior tibial and PAs. Bar graph showing the percentage of analyzed vessels (14 patients/14 limbs, 28 vessels total) within each calcification grade (0-4), with paired-vessel comparisons summarized in the Results.

For calcium detection, Alizarin Red S staining was performed on deparaffinized sections. MAC was graded semiquantitatively on a scale of 0 to 4 based on the circumferential extent and thickness of Alizarin Red-positive deposits within the tunica media, using a scoring schema consistent with prior validated approaches to vascular calcification burden.^3^, ^4^, ^5^, ^6^ Grade 0 indicated no MAC; grade 1, focal or speckled deposits occupying <25% of the medial circumference; grade 2, deposits occupying 25% to 50%; grade 3, deposits occupying 51% to 75%; and grade 4, near-circumferential dense MAC occupying >75% of the medial circumference. MAC grading was performed by two independent experts blinded to arterial origin (ATA vs PA) using the predefined semiquantitative 0 to 4 scoring system. Representative images across all grades are shown in Fig 3.

### Clinical variables and outcomes

Clinical data were abstracted from the electronic medical record by trained personnel masked to the histological findings. Variables included age, sex, self-reported ethnicity, DM status, most recent hemoglobin A1c (HbA1c) value, ESRD status and dialysis modality, smoking history (never vs former vs current), glucagon-like peptide-1 receptor agonist (GLP-1 RA) use (active prescription at time of amputation), Rutherford category at presentation, and type and date of index revascularization procedure (surgical bypass or endovascular therapy). Time from index revascularization to amputation was calculated in days for all patients who underwent a prior revascularization procedure, including the control without PAD, who underwent revascularization for acute arterial occlusion unrelated to PAD. When available, preoperative angiographic studies were reviewed qualitatively for infrapopliteal patency; amputated specimens were not imaged before histological sectioning.

### Definition and categorization of MAC burden

For vessel-level analyses, MAC burden was defined by the maximum Alizarin Red MAC grade across all available cross-sectional sections per limb. The within-patient concordance of MAC grades between paired ATAs and PAs was assessed using exact grade agreement and within-one-grade agreement as concordance metrics.

### Statistical analysis

This pilot study was designed for hypothesis generation and preliminary effect size estimation rather than definitive statistical inference. Analyses evaluated the concordance of MAC grades between paired ATAs and PAs and explored the associations with clinical variables including age, ethnicity, HbA1c, ESRD stage, GLP-1 RA use, smoking, and time to amputation from revascularization. Continuous variables are presented as mean ± standard deviation or median with interquartile range as appropriate based on distribution normality assessed by the Shapiro-Wilk test. Categorical variables are presented as counts and percentages. Pearson or Spearman correlation coefficients were calculated for exploratory analyses of MAC grade vs age, HbA1c, and Rutherford category, as appropriate. Analyses of HbA1c, GLP-1 RA use, ESRD stage, and Rutherford category excluded the control without PAD because these clinical factors were not relevant to this patient. Between-group comparisons of MAC grade were performed using the Wilcoxon rank-sum test for non-normally distributed ordinal data. A two-tailed *P* value of <.05 was considered statistically significant. Given the small sample size, all associations are reported as exploratory trends without inferential conclusions, and we explicitly acknowledge that this study is underpowered for multivariable modeling or formal survival analysis. Analyses were performed using JMP version 14.2 (SAS Institute).

## Results

### Patient characteristics

The final cohort comprised 13 patients with advanced PAD and 1 control without PAD, with clinical characteristics summarized in the Table. The PAD group included 92.3% male patients with average age of 69 ± 10 years and demonstrated a high prevalence of DM and ESRD, consistent with previously published PAD series enriched for MAC.^3^, ^4^, ^5^, ^6^ Smoking history and hypertension were common comorbidities. Many patients with PAD presented with Rutherford category 5 to 6 ischemia, indicating tissue loss or frank gangrene, and most ultimately required major amputation of the affected limb.

### Histological MAC patterns and grading

In paired analyses of ATA and PA segments, there was no significant difference in semiquantitative MAC grade between vessels (Fig 2, A). Percent intima and percent stenosis were also similar in the ATAs and PAs, with no systematic shift toward greater intimal remodeling or narrowing in either segment (Figs, 2, B, C). Although the PA had a smaller total vessel and luminal area compared with its paired ATA, the total vessel area and luminal area did not differ significantly between the ATAs and PAs (Figs 2, D, E), indicating comparable overall vessel sizes and preserved lumen across the distal tibial and peroneal beds. Taken together, these morphometric and calcification data suggest that, within a given limb, the ATA and PA exhibit broadly similar patterns of MAC and structural remodeling.

Alizarin Red staining revealed MAC in the majority of limbs with PAD, with MAC grades ranging from 0 to 4 across individual specimens. The semiquantitative 0 to 4 grading scale and representative photomicrographs are shown in Fig 3, illustrating the progression from absent to dense, near-circumferential MAC. At the limb level, the majority of PAD specimens, independent of arterial bed (ATA and PA), fell into the MAC-high category (grades 3-4), whereas the control without PAD demonstrated grade 0 MAC in both the TA and PA segments.

### ATA vs PA concordance

Fig 3, B, includes all 28 vessel specimens from the 14 patients and 14 limbs, with the MAC grade shown for each artery. Among patients with paired vessels, within-patient concordance of MAC grade was high, with 11 of 13 limbs (84.6%) showing exact agreement and all but one pair differing by only a single grade. The PA was not systematically spared; peroneal segments mirrored the MAC burden of the paired ATA within each limb. These observations establish MAC as a diffuse distal arterial phenotype driven predominantly by systemic factors such as DM and ESRD, rather than a focal, segment-specific process dictated by local hemodynamics.^1^^,^^3^, ^4^, ^5^ From a histological perspective, the high ATA-PA concordance suggests that MAC grading in a single infrapopliteal artery may reasonably index the limb-wide MAC burden in advanced PAD, supporting the use of standardized distal MAC metrics for risk stratification and for evaluating anticalcification strategies.

### MAC vs clinical risk factors

Exploratory analyses of MAC grade in relation to age, ethnicity, HbA1c, GLP-1 RA use, ESRD stage, and smoking status were performed. The MAC grade tended to be higher among patients with DM, advanced ESRD, and a history of tobacco use. GLP-1 RA use appeared to be more common in patients with a greater MAC burden, although this association was not statistically evaluated given the small sample size. All clinical associations showed directional trends in the expected direction, but did not attain statistical significance in this small pilot cohort.

### MAC vs Rutherford category and limb outcomes

**Fig 4:**
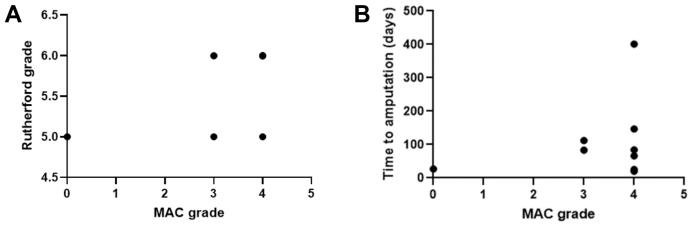
Medial arterial calcification (*MAC*) grade vs Rutherford category and limb outcomes. **(A)** Scatterplot of MAC grade stratified by Rutherford category at presentation. **(B)** Scatterplot of MAC grade vs time from index revascularization to major amputation (days). Each dot represents one patient; overlapping points indicate identical or similar values.

The relationship between MAC grade and Rutherford category at presentation, and the exploratory association between MAC grade and time to major amputation, are displayed in Fig 4. Limbs with higher Rutherford categories generally exhibited a higher MAC grade. Patients with a higher MAC grade tended to have shorter intervals from the index revascularization procedure to major amputation, although the number of events was insufficient for formal survival modeling. These directional findings are consistent with imaging-based studies linking infrapopliteal calcification burden with increased amputation and mortality risks.^2^^,^^10^

## Discussion

The principal finding of this pilot histopathological study is the high concordance of histological MAC burden between paired ATA and PA segments. This observation supports the concept that MAC in advanced PAD reflects a shared distal arterial phenotype rather than an isolated vessel-specific process. In practical terms, the similar MAC grades observed across the tibial and PAs suggest that distal medial calcification is distributed broadly within the limb, even when luminal disease and revascularization targets may differ. These findings extend prior histopathological comparisons of plaque characteristics between ATA and posterior TAs by specifically focusing on MAC as a vessel-level histological readout and by including the PA, a clinically important but undercharacterized below-knee segment.^1^

Historical pathology studies of amputated limbs described a differential distribution of lower extremity arterial disease, with the PA often appearing less affected by occlusive disease than the tibial vessels. However, these observations were based on gross and histological assessment of older amputated limb series rather than modern vessel-level MAC grading.^15^ In this study of human infrapopliteal arteries from patients with PAD, MAC emerged as a shared distal arterial phenotype across ATA and PA segments rather than a segment-specific lesion. High within-patient concordance of MAC burden across paired vessel segments supports the interpretation that distal MAC reflects systemic drivers of vascular calcification. Local hemodynamic and anatomical factors unique to individual tibial segments do not substantially determine MAC burden; instead, systemic metabolic disease appears to impose a shared calcification phenotype uniformly across the distal arterial bed.

Although complete imaging data were not available for all vascular beds, available clinical reports indicated that calcification outside the infrapopliteal circulation was common among patients with high tibial MAC. Among these patients, femoropopliteal calcification was present in 10 of 12 patients and aortoiliac calcification in 7 of the 12, supporting the possibility that the observed MAC burden reflects a broader systemic calcific vasculopathy. The finding that PA segments generally share the same MAC burden and degree of remodeling as ATA segments suggests that ATAs and PAs may share a common distal MAC phenotype in advanced PAD, warranting further study of vessel-level calcification patterns in larger cohorts. Our data also suggest that systemic drivers may impose a shared medial disease state across distal TAs and PAs, with angiographic patency differences, likely reflecting factors other than intrinsic protection from MAC. From a translational perspective, the close tibial-peroneal concordance in both calcification and morphometric measures supports the use of distal MAC indices as limb-level histological measures and implies that standardized assessment of a single infrapopliteal segment may reasonably approximate the medial disease burden across the distal arterial tree.

Prior imaging studies have shown that the distribution of infrapopliteal stenosis is not uniform across tibial vessels, and angiographic cohorts often report different patterns of ATA, posterior TA, and PA involvement.^16^^,^^17^ These findings address occlusive disease distribution rather than histological MAC burden, which was the focus of the present study. Available preoperative angiographic data provided only qualitative support for the histological findings. In several patients, infrapopliteal patency on angiography did not correspond with the degree of MAC observed histologically, particularly in the PA. For example, several limbs with severe histological MAC demonstrated preserved angiographic patency, whereas limbs with milder histological disease also showed vessel patency. However, amputated specimens were not imaged before sectioning, and imaging was not consistently available across the cohort. Accordingly, these observations should be interpreted as supportive but exploratory; future studies with standardized preoperative and ex vivo imaging are needed to define the relationship between angiographic appearance and histological MAC burden. Infrapopliteal disease often reflects a mixed phenotype in which medial calcification and occlusive disease coexist, and this combination has implications for endovascular therapy, lesion crossing, and procedural durability.^18^

Histological and pathobiological studies have long recognized medial calcification as a contributor to arterial stiffening and impaired vascular reactivity in PAD, particularly in patients with DM.^19^ The clustering of a high MAC burden with DM and advanced ESRD is consistent with prior literature, but the small sample size and cohort imbalance preclude inferences regarding independent associations.^3^, ^4^, ^5^, ^6^, ^7^, ^8^ These findings should therefore be interpreted as exploratory and hypothesis generating. The directional association between histological MAC burden and adverse limb outcomes in our cohort is consistent with prior imaging-based evidence linking infrapopliteal calcification with major amputation and mortality.^2^ Broader studies across vascular beds have also demonstrated that calcification burden is a robust predictor of major adverse cardiovascular events, independent of traditional risk factors.^9^^,^^10^ Vessel-level MAC quantification on human tissue may provide a biologically grounded correlate of radiographic calcification scores. Whether histological MAC burden correlates with clinical outcomes such as Rutherford category or predicts limb loss risk beyond imaging requires prospective evaluation in larger, clinically annotated cohorts.

### Limitations

This study has several important limitations. The sample size was small and derived from a single center, limiting generalizability and precluding formal multivariable adjustment or survival modeling. Quantitative morphometric measurements (total vessel area, luminal area, percent intima, and percent stenosis) could not be reliably obtained in the non-PAD control specimen because the arterial architecture was distorted in a manner that precluded consistent delineation of the intimal and medial boundaries. As a result, the morphometric and stenosis analyses are restricted to limbs with PAD and are primarily interpreted as within-limb and between-artery (ATA vs PA) comparisons, rather than as absolute differences between PAD and non-PAD tissue. As an amputation-based tissue study, the cohort is inherently enriched for terminal end-stage disease, introducing selection bias toward patients with the most advanced MAC. MAC grading was performed using a semiquantitative ordinal scale, which, although consistent with established approaches, is subject to interobserver variability; future studies should use morphometric image analysis to generate continuous calcification area measurements analogous to prior histopathological studies.^13^ Additionally, posterior tibial arteries were not systematically collected in the current protocol, limiting direct comparison with the prior report by Koyama et al.^13^

## Conclusions

Notwithstanding these limitations, the combination of paired ATA and PA specimens, multimodal histological characterization, and detailed clinical annotation provides a unique translational view of distal MAC in human PAD that is not available from imaging studies or autopsy series alone. These findings provide biologic validation of distal MAC patterning and support future studies integrating tissue-based and imaging-based assessments of calcification in PAD. This study should be interpreted as a small, amputation-based histological series that defines tissue-level MAC patterns in advanced PAD rather than as a study of clinical decision-making or procedural outcomes. The principal value of the data is the biologic characterization of distal calcification and remodeling across paired infrapopliteal vessels. The finding that PA segments generally share the same MAC burden and degree of remodeling as the ATA segments suggests that distal medial calcification is not restricted to a single infrapopliteal target vessel. This does not imply equivalence in revascularization utility, but rather indicates that the histological substrate of calcification is broadly shared across paired distal vessels in end-stage PAD. Larger studies integrating imaging, hemodynamics, and tissue histology are needed to determine how these patterns relate to clinical target selection and limb outcomes.

## Author Contributions

Conception and design: SA, JR, BE, AG

Analysis and interpretation: SA, JR, BE, AG

Data collection: SA, JR, AG

Writing the article: SA, JR

Critical revision of the article: SA, JR, BE, AG

Final approval of the article: SA, JR, BE, AG

Statistical analysis: SA, JR

Obtained funding: BE, AG

Overall responsibility: AG

SA and JR contributed equally to this article and share co-first authorship.

## Funding

Supported by the 10.13039/100007911UCSD Department of Surgery and Department of Vascular and Endovascular Surgery.

## Disclosures

None.

## References

[1] Fowkes F.G.R., Rudan D., Rudan I. (2013). Comparison of global estimates of prevalence and risk factors for peripheral artery disease in 2000 and 2010: a systematic review and analysis. Lancet.

[2] Zettervall S.L., Marshall A.P., Fleser P., Guzman R.J. (2018). Association of arterial calcification with chronic limb ischemia in patients with peripheral artery disease. J Vasc Surg.

[3] Lanzer P., Boehm M., Sorribas V. (2014). Medial vascular calcification revisited: review and perspectives. Eur Heart J.

[4] Lanzer P., Hannan F.M., Lanzer J.D. (2021). Medial arterial calcification: JACC state-of-the-art review. J Am Coll Cardiol.

[5] Demer L.L., Tintut Y. (2008). Vascular calcification: pathobiology of a multifaceted disease. Circulation.

[6] Johnson R.C., Leopold J.A., Loscalzo J. (2006). Vascular calcification: pathobiological mechanisms and clinical implications. Circ Res.

[7] Durham A.L., Speer M.Y., Scatena M., Giachelli C.M., Shanahan C.M. (2018). Role of smooth muscle cells in vascular calcification: implications in atherosclerosis and arterial stiffness. Cardiovasc Res.

[8] Lee S., Woo J., Chen Y., Arya S., Allison M. (2026). Peripheral vascular calcification. Arterioscler Thromb Vasc Biol.

[9] Dong Y., Liu Y., Cheng P. (2023). Lower limb arterial calcification and its clinical relevance with peripheral arterial disease. Front Cardiovasc Med.

[10] Liu I.H., Wu B., Krepkiy V. (2022). Pedal arterial calcification score is associated with the risk of major amputation in chronic limb-threatening ischemia. J Vasc Surg.

[11] Kim T.I., Guzman R.J. (2023). Medial artery calcification in peripheral artery disease. Front Cardiovasc Med.

[12] Allison M.A., Hsi S., Wassel C.L. (2012). Calcified atherosclerosis in different vascular beds and the risk of mortality. Arterioscler Thromb Vasc Biol.

[13] Koyama Y., Migita S., Shimodai-Yamada S. (2023). Pathology of critical limb ischemia; comparison of plaque characteristics between anterior and posterior tibial arteries. J Atheroscler Thromb.

[14] Bishop P.D., Feiten L.E., Ouriel K. (2008). Arterial calcification increases in distal arteries in patients with peripheral arterial disease. Ann Vasc Surg.

[15] Ferrier T.M. (1967). Comparative study of arterial disease in amputated lower limbs from diabetics and non-diabetics (with special reference to feet arteries). Med J Aust.

[16] Wikström J., Hansen T., Johansson L., Ahlström H., Lind L. (2009). Lower extremity artery stenosis distribution in an unselected elderly population and its relation to a reduced ankle-brachial index. J Vasc Surg.

[17] Ramdass M.J., Harnarayan P., Mooteeram N. (2014). Patterns of arteriosclerotic lesions of the lower extremity in a West Indian population based on angiographic findings and ethnicity. Ann R Coll Surg Engl.

[18] Mustapha J.A., Diaz-Sandoval L.J., Saab F. (2017). Infrapopliteal calcification patterns in critical limb ischemia: diagnostic, pathologic and therapeutic implications in the search for the endovascular holy grail. J Cardiovasc Surg (Torino).

[19] Soor G.S., Vukin I., Leong S.W., Oreopoulos G., Butany J. (2008). Peripheral vascular disease: who gets it and why? A histomorphological analysis of 261 arterial segments from 58 cases. Pathology.

